# Biochemical prevention and treatment of viral infections – A new paradigm in medicine for infectious diseases

**DOI:** 10.1186/1743-422X-1-12

**Published:** 2004-11-23

**Authors:** Hervé Le Calvez, Mang Yu, Fang Fang

**Affiliations:** 1 Abgent, Inc. 6310 Nancy Ridge Drive, Suite 106, San Diego, CA 92121 USA; 2 NexBio, Inc. 6330 Nancy Ridge Drive, Suite 105, San Diego, CA 92121 USA

**Keywords:** viral mRNA, anti-sense oligonucleotide, ribozyme, RNA interference, viral infectious disease, blocking antibody, soluble receptor, rhinovirus

## Abstract

For two centuries, vaccination has been the dominating approach to develop prophylaxis against viral infections through immunological prevention. However, vaccines are not always possible to make, are ineffective for many viral infections, and also carry certain risk for a small, yet significant portion of the population. In the recent years, FDA's approval and subsequent market acceptance of Synagis, a monoclonal antibody indicated for prevention and treatment of respiratory syncytial virus (RSV) has heralded a new era for viral infection prevention and treatment. This emerging paradigm, herein designated "Biochemical Prevention and Treatment", currently involves two aspects: (1) preventing viral entry via passive transfer of specific protein-based anti-viral molecules or host cell receptor blockers; (2) inhibiting viral amplification by targeting the viral mRNA with anti-sense DNA, ribozyme, or RNA interference (RNAi). This article summarizes the current status of this field.

## Introduction

**Figure 1:**
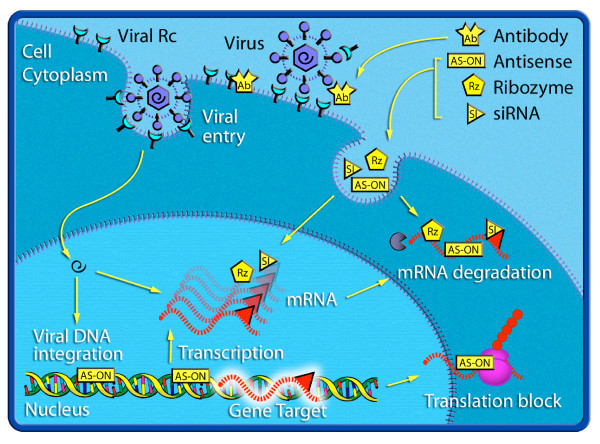
**Targets of different Biochemical Prevention and Treatment strategies**. Antibodies (Ab) or soluble receptors (Rc) can inhibit the viral entry. Antisense oligonucleotides (AS-ONs), ribozymes (Rz) or siRNA (SI) pair with their complementary target genomic DNA, RNA or mRNA. AS-ONs can block recombination, transcription, translation of the mRNA or induce its degradation by RNaseH. Rz possess catalytic activity and cleave their targets. SiRNAs (SI) induce degradation of the target mRNA via RNA-induced silencing complex (RISC).

A landmark in the battle against viral infectious diseases was made in 1798 when Jenner first inoculated humans against smallpox with the less virulent cowpox. For about two centuries since then, humans relied almost exclusively on vaccines for protection against viruses. Only in the recent years, new strategies for controlling viral infectious diseases have emerged, which have so far led to a couple of viral prophylaxis/therapeutics on the market. These strategies are fundamentally different from vaccines in that they attempt to directly interrupt viral infectious life cycle at molecular level by using proteins or oligonucleotides. To differentiate them from the conventional vaccines that prevent viral infection by boosting immune system, we refer the new antiviral approaches as "Biochemical Prevention and Treatment" (see figure 1). Biochemical Prevention and Treatment, as an alternative to vaccines and chemical compound based antiviral drugs, may prove to be particularly valuable in the areas where vaccines and/or chemical drugs can not be generated or have not been successful in human, including diseases caused by some common pathogenic viruses, such as HIV, hepatitis C virus (HCV), RSV and human rhinovirus (HRV). In this review, we will discuss various molecular intervention approaches.

### 1. Biochemical Prevention and Treatment via Protein targeting

Among the biochemical therapeutics currently in clinical trials, the majority consists of monoclonal antibodies (MAbs). Soluble receptor drug candidates have gradually lost favor over the past several years due to issues relating to low potency and cost. Peptide-based drug candidates are limited by insufficient efficacy and unfavorable pharmacokinetics. MAbs have increasingly gained favor in large part because of the development of chimeric, humanized, and human antibodies have reduced the immunogenicity of antibody therapies. The MAbs that are currently in clinical trials for viral infection prophylaxis and treatment are listed in Table 1.

**Table 1:** Monoclonal Antibodies in Clinical Trials

**Product**	**Company**	**Disease**	**Status**
MEDI-501	MedImmune	Genital Warts HPV	II
Nabi-HB	Nabi Biopharmaceuticals	Hepatitis B	Market
Ostavir	Protein Design Labs	Hepatitis B	II
XTL-002	XTL Biopharmaceuticals Ltd.	Hepatitis C	I
Civacir	Nabi Biopharmaceuticals	Hepatitis C	I/II
1F7 Antibody	Immune Network Ltd.	Hepatitis C, HIV/AIDS	Preclinical
PRO 140	Progenics Pharmaceuticals	HIV/AIDS	Preclinical
hNM01	AbNovo Inc., Immune Network Ltd.	HIV/AIDS	I
PRO 367	Roche Holding Progenics Pharmaceuticals	HIV/AIDS	I/II
TNX-355	Tanox, Inc., Biogen, Inc. (Massachusetts)	HIV/AIDS	I
OraQuick HIV-1	OraSure Technologies, Inc.	HIV/AIDS	Market
Cytolin	CytoDyn Amerimmune Pharmaceuticals, Inc.	HIV/AIDS	I/II
Tipranavir	TIPRANAVIR	HIV/AIDS	III
HXB	AAI International, AnaaiPharma Company	Herpes Simplex Virus type 2	Preclinical
MEDI-491	MedImmune	Human B19 parvovirus	I
Synagis™ (Palivizumab)	MedImmune	Respiratory Syncytial Virus	Approved in 1998
Numax	MedImmune	Respiratory Syncytial Virus	Preclinical
INS37217 Intranasal	Inspire Pharmaceuticals	Rhinovirus (common cold)	II

#### Biochemical Prevention and Treatment of Respiratory Syncytial Virus Infection

The respiratory syncytial virus (RSV) is a major cause of lower respiratory tract infection in infants and young children producing bronchiolitis and pneumonia worldwide. RSV infection leads to more than 90,000 hospitalizations and a 2% mortality rate among infants nationwide [2-5]. Approximately two-thirds of infants are infected with RSV during the first year of life and approximately 95% of children test seropositive for RSV by the age of two [6]. Unfortunately, even natural RSV infection produces limited immunity at best. In fact, an inactivated RSV vaccine paradoxically resulted in more severe disease instead of protection [7].

The most successful approach to date has been Biochemical Prevention and Treatment with anti-viral antibodies. In 1996, RespiGam™ (respiratory syncytial virus immune globulin or RSV-IG) became available for use in children less than two years of age with high-risk factors [8-10]. The use of RespiGam™ was largely supplanted with the approval of Synagis™ (Palivizumab) in 1998. Palivizumab is an IgG1 MAb administered IM monthly that selectively binds to the RSV surface glycoprotein F [1,51]. The drug specifically inhibits RSV replication by preventing the virus from fusing with the respiratory endothelial cell membrane. Palivizumab has been shown to reduce the rate of hospitalization of at-risk infants by about 55% in clinical studies and now serves as the primary medical means of RSV prevention [11-13].

#### Prevention of Human Rhinovirus infections

Human rhinovirus (HRV) causes over 80% of the common cold in the fall [14]. Developing vaccines against HRV is unfeasible because HRVs have at least 115 antigenically distinct serotypes [15,16]. One of the proven methods to prevent and inhibit viral infections is to block host cell receptors that are used by viruses to gain cell entry. Receptor blockage is commonly achieved via application of MAbs that bind to specific epitopes on the receptor molecules. A plethora of *in vitro *studies have reported effective viral inhibition by receptor-blocking MAbs. However, these works have not yielded yet any approved drug on the market.

In HRV infection, about 90% of HRV serotypes utilize a single cell surface receptor exclusively, which is the intercellular adhesion molecule-1 (ICAM-1), for viral attachment and subsequent viral entry [17,18]. As such, ICAM-1 has become a very promising target for biochemical prevention. A receptor blocking approach has shown that the soluble ICAM-1 and an anti-ICAM-1 monoclonal antibody, Mab 1A6, could prevent infections by a broad spectrum of rhinovirus serotypes in human cells *in vitro *[19-21]. Administration of soluble ICAM-1 and MAbs in human clinical trials had indeed achieved reduction in symptoms, but did not prevent the incidence of the disease [22-24]. For the MAbs, the limited efficacy is most likely due to its low functional affinity (or avidity) for ICAM-1 when compared to that of the multivalent HRV particles [25].

High avidity is achieved by multivalency. To improve avidity of HRV receptor blocking antibody, a novel tetravalent recombinant antibody, CFY196, has been generated against ICAM-1 [26]. CFY196 is composed of Fab fragment of a humanized version of MAb 1A6 fused with a linker derived from human immunoglobulin D (IgD) hinge and a tetramerization domain derived from the coiled-coil sequence of human transcription factor ATFα. CFY196 is expressed in bacteria and purified as a homogenous tetrameric molecular complex. CFY196 exhibited almost two-orders-of-magnitude improvement in functional affinity compared with its bivalent counterpart based on the kinetic parameters measured by BIAcore analysis. Such kinetic improvement also directly leads to functional superiorities of CFY196. In *in vitro *assays, CFY196 consistently and significantly outpaced the best commercial anti-ICAM-1 MAbs in preventing HRV infection as measured by reduction of cytopathic effects and HRV viral titers [26]. The preclinical findings of CFY196 bode well its efficacy in human since MAb 1A6, from which CFY196 is derived, has already exhibited positive effects in a human trial. Moreover, to prevent possible immunogenicity, CFY196 is humanized [27]. Further pre-clinical and clinical development of CFY196 is warranted to fully evaluate its potential as a prophylaxis and therapeutics for the HRV induced common colds.

### 2. Biochemical Prevention and Treatment via targeting on viral mRNA

Targeting viral mRNA is one of the most active areas of research and development. Several strategies have emerged over the years and are being tested pre-clinically and clinically. They include: antisense-oligonucleotides (AS-ONs), ribozymes, and recently, RNA interference (RNAi). All these strategies share the features of conceptual simplicity, straightforward drug design and quick route to identify drug leads. However, the challenges have been to improve potency, pharmacokinetics and, most importantly, intracellular delivery of the drug candidates. As the oldest strategy, AS-ON technology has produced to date one drug in the market place, Vitravene^®^. A number of clinical trials of drug candidates from these technologies are currently ongoing.

#### Antisense-oligonucleotides

Antisense-oligonucleotides (AS-ONs) are short synthetic oligonucleotides that form complementary pair with specific viral mRNA targets. AS-ONs inhibit viral protein production by both blocking viral mRNA translation and triggering its degradation. Since the discovery of viral inhibition effect of AS-ONs by Zamecnik and Stephenson in 1978 [28], antisense technology has been developed as a powerful tool for target validation and therapeutic purposes.

Vitravene is the first AS-ON based drug approved by FDA. Vitravene, or fomivirsen sodium, is a 21-base phosphorothioate oligodeoxynucleotide complementary to the messenger RNA of the major immediate-early region proteins of human cytomegalovirus, and is a potent and selective antiviral agent for cytomegalovirus retinitis, a herpes-like eye disease that afflicts the immune-suppressed [29,30]. A number of clinical trials as well as one approved therapy based on AS-ON technologies are summarized in Table 2.

**Table 2:** Clinical trials and an approved therapy based on AS-ON technologies [31-33].

**Product**	**Company**	**Target**	**Disease**	**Chemistry**	**Status**
Vitravene (Fomivirsen)	ISIS Pharmaceuticals	CMV IE2	CMV retinitis	PS DNA	Approved in 1998
Affinitac (ISIS 3521)	ISIS	PKC-α	Cancer	PS DNA	Phase III
Genasense	Genta	Bcl2	Cancer	PS DNA	Phase III
Alicaforsen (ISIS 2302)	ISIS	ICAM-1	Psoriasis, Crohn's disease, Ulcerative colitis	PS DNA	Phase II/III
ISIS 14803	ISIS	Antiviral	Hepatitis C	PS DNA	Phase II
ISIS 2503	ISIS	H-ras	Cancer	PS DNA	Phase II
MG98	Methylgene	DNA methyl transferase	Solid tumors	PS DNA	Phase II
EPI-2010	EpiGenesis Pharmaceuticals	Adenosine A1 receptor	Asthma	PS DNA	Phase II
GTI 2040	Lorus Therapeutics	Ribonucleotide reductase (R2)	Cancer	PS DNA	Phase II
ISIS 104838	ISIS	TNFα	Rheumatoid Arthritis, Psoriasis	2nd generation	Phase II
Avi4126	AVI BioPharma	c-myc	Restenosis, cancer, Polycystic kidney disease	3rd generation	Phase I/II
Gem231	Hybridon	PKA RIα	Solid tumors	2nd generation	Phase I/II
Gem92	Hybridon	HIV gag	AIDS	2nd generation	Phase I
GTI 2051	Lorus Therapeutics	Ribonucleotide reductase (R1)	Cancer	PS DNA	Phase I
Avi4557	AVI BioPharma	CYP3A4	Metabolic redirection of approved drugs	3rd generation	Phase I

Phosphorothioate (PS) oligodeoxynucleotides are the '*first generation*' DNA analogs. The '*second generation*' ONs contain nucleotides with alkyl modifications at the 2' position of the ribose. They are less toxic than PS-DNAs and have a slightly enhanced affinity. DNA and RNA analogs with modified phosphate linkages, or different sugar residues substituting the furanose ring have been referred as '*third generation*' [34]. For instance, peptide nucleic acids and their analogs display superior sequence specificity and are resistant to nuclease degradation. These third generation AS-ON have limited non-specific interactions with other genes and, therefore, have shown great potentials in clinical trials.

#### Ribozymes

Ribozymes (Rz) are catalytically active ONs that both bind and cleave target RNAs. They were discovered after the AS-ON technology. Initial findings on ribozymes raised the hope that they may offer a more potent alternative to AS-ONs. Many cell based and animal tests have performed on anti-viral effects of ribozymes, including HIV, hepatitis B, hepatitis C, influenza, etc. Results from these tests have shown that ribozymes are promising viral inhibitors [35-38]. However, further progress in the field has been hampered by difficulties to achieve satisfactory potency and efficient intracellular delivery of ribozymes in vivo. HEPTAZYME is a modified ribozyme that cleaves the internal ribosome entry site of the Hepatitis C virus. The Rz was demonstrated to inhibit viral replication up to 90% in cell culture [39]. HEPTAZYME was tested in a Phase II clinical trial, but was later withdrawn from further clinical trials due to insufficient efficacy. So far, there is no anti-viral ribozymes that are being actively tested in advanced clinical trials.

#### RNA Interference (RNAi)

RNA interference, or RNAi, is the inhibition of expression of specific genes by double-stranded RNAs (dsRNAs). It is becoming the method of choice to knockdown gene expression rapidly and robustly in mammalian cells. Comparing to the traditional antisense method, RNAi technology has the advantage of significantly enhanced potency; therefore, only lower concentrations may be needed to achieve same level of gene knockdown. RNAi gained rapid acceptance by researchers after Tuschl and coworkers discovered that *in vitro *synthesized small interfering RNAs (siRNAs) of 21 to 23 nucleotides in length can effectively silence targeted genes in mammalian cells without triggering interferon production [40,41]. In mammalian cells, the level of gene inhibition mediated by siRNA routinely reaches an impressive 90% [42].

Several initial studies, which test the potential application of synthetic siRNAs as antiviral agents, have shown very promising results. To date, RNAi has been used effectively to inhibit the replication of several different pathogenic viruses in culture, including: RSV (respiratory syncytial virus) [43], influenza virus [44], poliovirus [45] and HIV-1 [46-48]. In the case of HIV-1, several specific mRNAs have been successfully targeted for siRNA-mediated silencing, including those that encode Gag, Pol, Vif and the small regulatory proteins Tat and Rev. These studies show that RNAi can effectively trigger the degradation of not only viral mRNAs, but also genomic RNAs at both the pre- and post-integration stages of the viral lifecycle. In addition to targeting viruses directly, alternative strategies have employed siRNAs that silence the expression of essential host factors including Tsg101, required for vacuolar sorting and efficient budding of HIV-1 progeny [49], and the chemokine receptor CCR5, required as a co-receptor for HIV-1 cell entry [50].

## Conclusions

Currently, our understanding of the biological mechanisms underlying RNAi lags behind the movement to apply this technology to human diseases such as viral infections. Some major technical hurdles need to be overcome before siRNA-based anti-viral prophylaxis and treatments move into the clinics. Especially, intracellular delivery of siRNA needs to be greatly improved. The next few years of research will indicate whether RNAi technology will realize its potential as the next wave of Biochemical Prevention and Treatment.

## Competing Interests

Dr. Hervé Le Calvez declares that he has no competing interest. Dr. Mang Yu and Dr. Fang Fang are the co-founders and current share holders of Perlan Therapeutics who has developed CFY196.

**Figure 2:**
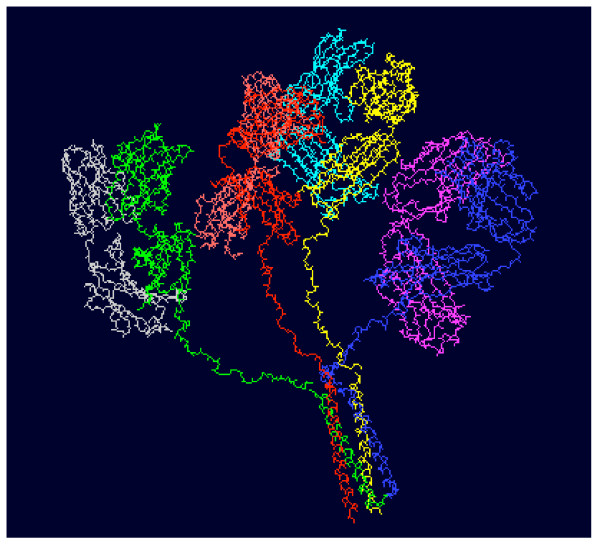
3D model of the tetrameric Fab anti-ICAM-1 molecule CFY196 [26]. Each identical subunit is represented by a different color.
